# Hematology of Wild Caught Dubois's Tree Frog *Polypedates teraiensis*, Dubois, 1986 (Anura: Rhacophoridae)

**DOI:** 10.1155/2014/491415

**Published:** 2014-01-30

**Authors:** Madhusmita Das, Pravati Kumari Mahapatra

**Affiliations:** Cell and Developmental Biology Laboratory, P.G. Department of Zoology, Utkal University, Bhubaneswar, Odisha 751 004, India

## Abstract

Blood was analyzed from eighty (forty males and forty females) adult individuals of *Polypedates teraiensis* to establish reference ranges for its hematological and serum biochemical parameters. The peripheral blood cells were differentiated as erythrocytes, lymphocytes, eosinophils, neutrophils, monocytes, basophils, and thrombocytes, with similar morphology to other anurans. Morphology of blood cells did not vary according to sex. The hematological investigations included morphology and morphometry of erythrocytes, morphometry of leucocytes, packed cell volume (PCV), hemoglobin content (Hb), mean corpuscular volume (MCV), mean corpuscular hemoglobin (MCH), mean corpuscular hemoglobin concentration (MCHC), erythrocyte or red blood cell (RBC) count, leukocyte or white blood cell (WBC) count, differential leukocyte count, and neutrophil to lymphocyte (N/L) ratio. Besides, protein, cholesterol, glucose, urea, uric acid, and creatinine content of blood serum were assayed. Hematological parameters that differed significantly between sexes were RBC count, length and breadth of RBC, neutrophil %, N/L ratio, area occupied by basophils, and diameter of large lymphocyte and eosinophils. The level of glucose, urea, and creatinine in blood serum also significantly differed between sexes.

## 1. Introduction

Hematology is the most straightforward and less invasive technique to access the health status of natural population of vertebrates [1]. These parameters allow the detection of changes in physiological, pathological, ecological, and environmental conditions in natural population [2, 3]. Differential leucocyte count provides information about the immunological status of an individual. Similarly, hematocrit reflects the efficiency of oxygen carrying capacity. The plasma or serum biochemical analyses provide information about internal organs, electrolytes, proteins, and nutritional and metabolic parameters [4]. Amphibians are known to be sensitive animals and show physiological variables to acute environmental changes [5]. For this reason it has been suggested that physiological ecology of these animals should be incorporated into conservation plans and monitoring programs of individual populations [6]. But hematological and serum biochemical reference ranges exist for most of the animal species that receive veterinary care [4]. Though there are many hematological reports on anuran species, there is less information available for the rhacophorid species [7, 8]. Detailed hematological reports on rhacophorid frog, *Polypedates maculatus,* have been studied by Mahapatra et al. [8]. Another frog that lives sympatric with *P. maculatus* is *P. teraiensis* which is arboreal and mostly found in bushes, plantations, and gardens and rarely enters human habitation [9]. Blood cell profile of the tadpoles of *P. teraiensis* was described by us earlier [10] and here we describe the hematological and serum biochemical parameters of adults. 

## 2. Materials and Methods

**Figure 1:**
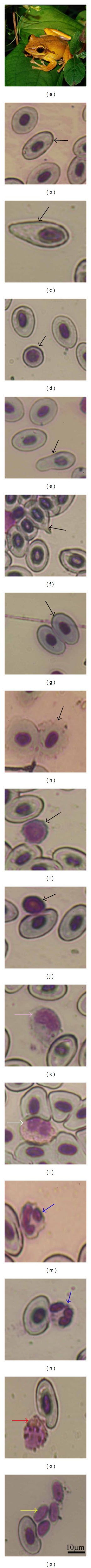
(a) Adult *Polypedates teraiensis*, (b) elliptical RBC with centrally placed nucleus, (c) elliptical RBC with eccentrically placed nucleus, (d) circular RBC with centrally placed nucleus, (e) spindle-shaped RBC, (f) teardrop-shaped cell, (g) dividing erythrocytes, (h) destruction of erythrocytes' membrane, (i) large lymphocyte, (j) small lymphocyte, (k) monocyte, (l) eosinophil, (m) tetralobed neutrophil, (n) trilobed neutrophil, (o) basophil, and (p) cluster of thrombocytes (scale bar = 10 *μ*m, (b)–(p)).

Adults of *Polypedates teraiensis* (Figure 1(a)) were collected from Chowduar (20°31′11′′N, 85°49′11′′E), Odisha. Forty adult specimens from each sex of the species were utilized in the present investigation. After collection, the frogs were maintained in the terrarium for acclimatization to laboratory conditions and handled following standardized procedures [11]. Blood samples were taken from the ventral abdominal vein. Prior to blood collection, specimens were weighted (in grams) and snout to vent length (SVL) was measured (in cm). Blood was collected in the morning hours to avoid diurnal variation. Collected blood was transferred from the syringe to a penicillin vial and kept undisturbed for clotting. After following retraction of clot, the supernatant serum was pipetted into an eppendorf tube. The serum was then used for all biochemical investigations. For other hematological investigations the collected blood was transferred from the syringe to a penicillin vial containing pinch of ethylene diamine tetra acetic acid (EDTA), an anticoagulant. The blood was mixed well without frothing. Hematological investigations included morphology and morphometry of erythrocytes, morphometry of leucocytes, packed cell volume (PCV), hemoglobin content (Hb), mean corpuscular volume (MCV), mean corpuscular hemoglobin (MCH), mean corpuscular hemoglobin concentration (MCHC), erythrocyte or red blood cell (RBC) count, leukocyte or white blood cell (WBC) count, differential leukocyte count, and neutrophil to lymphocyte (N/L) ratio. Serum biochemical parameters included estimation of total protein, cholesterol, glucose, urea, uric acid, and creatinine content.

For morphology and morphometry of blood cells, blood smears were prepared using push slide technique. The dried blood smears were stained with Giemsa's stain and observed under light microscope (Hund H500 WETZLAR). Different types of blood cells present in the smear were identified following Turner [12], and Heatley and Johnson [13]. Slides were viewed in zigzag pattern, covering all parts of the blood smear and leukocytes were counted in each field of view until 100 cells were counted. Blood cells were photographed with the help of a Canon EOS 450 12.2 Mega pixel camera (EF-S 18-55 1S Kit) and measured using an ocular micrometer which was standardized against a stage micrometer (ERMA, Japan made). Formulae used by Arserim and Mermer [14] were followed for measurement of blood cells. For the other hematological investigations, procedures described by Coppo et al. [15] were followed. 

Chemicals used for the serum biochemical studies were of analytical grade. Bovine serum albumin (BSA) was obtained from SIGMA Chemicals Co., USA. Folin-phenol reagent was obtained from Sisco Research Laboratory, Mumbai, India. Eco-pak glucose kit, autozyme urea enzymatic kit, infinite liquid uric acid kit, and autozyme creatinine kit were procured from Accurex Biomedical Private Limited, Mumbai, India. All other chemicals were of the highest purified grade available. For the estimation of total protein and total cholesterol, regular laboratory methods, that is, those of Lowry et al. [16] and Rosenthal et al. [17], were followed, respectively. For the estimation of glucose, urea, uric acid, and creatinine, the abovementioned laboratory kits were used.

Statistical analysis was done following Kolmogorov-Smirnov test, *P* > 0.05; averages were compared with student's *t*-test. The significance level was *P* ≤ 0.05. Statistical analyses were performed by SPSS (version 10.00).

## 3. Results and Discussion

In the present study, SVL (snout to vent length) in males ranged from 6.0 to 7.0 cm with a mean of 6.42 ± 0.43 cm and in females it ranged from 6.0 to 9.2 cm with a mean of 7.47 ± 1.36 cm. In case of males the average body weight was 16.87 ± 0.62 g (ranged from 16.0 to 17.5 g) and in females average body weight was 19.37 ± 1.93 g (ranged from 17.5 to 21.5 g). Hematological and biochemical parameters investigated in the present study are represented in Tables 1 and 2.

### 3.1. Morphology of Blood Cell

The characteristics shape of amphibian erythrocytes is elliptical form [18]. Apart from the characteristic ellipsoidal shape, amphibians are also known to display wide variation in erythrocyte morphology across species [7, 19, 20]. In the present study, erythrocytes of various shapes were observed. The different shapes included elliptical cells with centrally placed nuclei (Figure 1(b)), elliptical cells with eccentrically placed nuclei (Figure 1(c)) and circular cells with centrally placed nuclei (Figure 1(d)). Ellipsoidal erythrocytes have also been reported in the blood smear of *Polypedates maculatus *[8]. Besides, some irregular forms such as spindle-shaped cells (Figure 1(e)) and teardrop-shaped cells (Figure 1(f)) were observed. Such irregular cells were also observed in the tadpole of this species and it was attributed to anemic state [10]. Dividing erythrocytes (Figure 1(g)) and erythrocytes with damaged cell membrane (Figure 1(h)) were also evident in the present study.

The lymphocytes observed were of two distinct forms, that is, large lymphocytes (Figure 1(i)) and small lymphocytes (Figure 1(j)). Both lymphocytes were rounded in shape. However, there was difference in size. The nuclei were rounded in shape both in large and small lymphocytes and occupied the entire cell leaving a narrow rim of light violet cytoplasm towards the periphery. Monocytes were rounded in shape and had eccentrically placed indented nuclei (Figure 1(k)). Eosinophils were also rounded in shape with segmented nuclei. Eosinophils had bilobed nuclei, located at one end of the cells (Figure 1(l)). Neutrophils were large and rounded in shape. They were easily distinguished from other cells due to their lobular and segmented nuclei. Neutrophils with tetralobed nuclei (Figure 1(m)) or trilobed nuclei (Figure 1(n)) were more common. Basophils investigated in this species were round cells having large dark violet-stained granules over the irregular nuclei as well as entire cells (Figure 1(o)). Thrombocytes or platelets were long and oval in shape having large and ovoid nuclei. They were found in clusters on the blood smears (Figure 1(p)). Earlier reports suggest amphibian thrombocytes to be nucleated and spindle-shaped [14].

### 3.2. Morphometry of Blood Cells

Length and breadth of erythrocytes remained 16.87 ± 1.37 *μ*m and 8.95 ± 0.12 *μ*m in males and 19.77 ± 1.53 *μ*m and 8.62 ± 0.34 *μ*m in females, respectively. The mean length of erythrocytes in females was more than the males and the difference was statistically significant (*t* = 2.922, df = 6, *P* = 0.026). Arserim and Mermer [14] have reported larger erythrocytes in case of females (23.03 *μ*m, 14.59 *μ*m) than males (22.32 *μ*m, 13.65 *μ*m) in *Rana macrocnemis*. The mean length and breadth of erythrocytes in both males and females were found to be less when compared to other Rhacophorid anurans, that is, *Buergeria buergeri* (19.8 *μ*m, 13 *μ*m), *Rhacophorus annamensis *(20 *μ*m, 12.7 *μ*m), and *Rhacophorus schlegelii* (21.6 *μ*m, 13.3 *μ*m) as reported by Kuramoto [7]. The aspect ratio (Length/breadth of RBC) was more in females than males. This was also statistically significant (*t* = 2.65, df = 6, *P* = 0.03). The surface area occupied by the erythrocytes was more in females than in males (Table 1), but this difference was statistically insignificant. But higher surface area of erythrocytes in males (243.15 ± 37.841 *μ*m^2^) in comparison to females (210.58 ± 38.279 *μ*m^2^) has been reported in adult frogs of *Polypedates maculatus* [8]. The mean value of long axes and short axes of nuclei remained 6.15 ± 0.264 *μ*m and 5.0 ± 0.08 *μ*m in males and 7.5 ± 1.73 *μ*m and 5.5 ± 0.90 *μ*m in females, respectively. The surface area of nuclei ranged from 23.01 to 25.85 *μ*m^2^ with a mean of 23.98 ± 1.27 *μ*m^2^ in case of males. In females it ranged from 20.7 to 49.64 *μ*m^2^ with a mean of 33.0 ± 12.80 *μ*m^2^. However, there was no significant difference between sexes for the parameters studied on nuclei of RBCs. 

The erythrocytes were larger in size in tadpoles of this species [10] in comparison to the adults of the present study. Snyder and Sheafor [21] have suggested that with the evolutionary development of more efficient cardiovascular system, and that they are attended by higher vascular resistances, including smaller capillary radial dimensions which are attended by smaller RBCs. Thus, decrease in size of RBCs in adult frogs of the present study in comparison to tadpoles [10] is an example of physiological adaptation required for transition from aquatic to terrestrial mode of life.

Knowledge on erythrocytes in vertebrates provides us with much valuable information. Snyder and Sheafor [21] have described erythrocytes to be the center piece in the evolution of vertebrate circulatory system. The measurement of erythrocyte dimensions is often an important component of standard hematologic survey in amphibians [22]. It can be used for comparison across species [23] and studies of environmental, seasonal, or altitudinal acclimatization [24–26]. Measuring erythrocytes can also provide information regarding the genome size of a species [7, 27]. In amphibians, erythrocyte has long been known to be correlated negatively with metabolic rates, both at the organism level [19, 28] and the tissue level [29]. This relationship stems from the fact that larger surface-area-to-volume ratios in smaller cells allow for more efficient exchange of oxygen. This idea is exemplified in intraspecific comparisons of amphibians at different altitudes, where animals at higher latitudes have smaller erythrocytes [24, 30], presumably to maximize cellular efficiency of oxygen transport and exchange in a low-oxygen environment. Some investigators have stressed that erythrocyte may be used in ploidy determination [31–34]. Moreover, erythrocyte size can also be used as a diagnostic assay to assess the effects of air pollution in animals [35].

Amongst the leucocytes, mean diameter of large lymphocytes, small lymphocytes, monocytes, and basophils remained more in males than in females (Table 1). The size difference was significant for large lymphocytes only (*t* = 2.26, df = 18, *P* = 0.036). The diameter of eosinophils was more in females (12.0 ± 0.31) than in males (11.5 ± 0.45) and the difference was significant (*t* = 2.89, df = 18, *P* = 2.893). There was no difference in mean size of neutrophils in both sexes. The area occupied by the large lymphocytes, small lymphocytes, basophils, and monocytes was more in males than in females. The area occupied by eosinophils was more in females than in males. There was no difference in mean area of neutrophils in both sexes (Table 1). The difference was significant for the area of basophils (*t* = 2.342, df = 18, *P* = 0.0309), while for other leucocytes the difference remained insignificant. The diameter of neutrophils (16.98 *μ*m), basophils (13.69 *μ*m), eosinophils (16.30 *μ*m), and monocytes (14.30 *μ*m) was more in *Rana macracnemis* [14] than in the present rhacophorid studied. Similarly, larger leucocytes (neutrophils = 15.2 *μ*m, lymphocytes = 13.6 *μ*m, eosinophils = 16.2 *μ*m, monocytes = 15.2 *μ*m, and basophils = 16.9 *μ*m) have been reported in *R. catesbeiana* by Coppo et al. [15].

### 3.3. RBC Count, Hemoglobin Concentration, and Red Blood Cell Indices

The mean number of RBCs in males was 0.59 ± 0.012 millions/mm^3^ of blood but in females it was 0.62 ± 0.017 millions/mm^3^ of blood. The number of RBCs remained more in females than in males. This difference was statistically significant (*t* = 4.2, df = 6, *P* = 0.005). The gram percentage of hemoglobin per 100 mL of blood varied from 5.8 to 6.1 with a mean of 5.95 ± 0.12 among males. In females it ranged from 5.5 to 6.2/100 mL with a mean of 5.82 ± 0.29/100 mL. The mean packed cell volume (PCV) was more in females (51.55 ± 0.95) than in males (50.625 ± 1.10) (Table 1). The mean corpuscular volume (MCV), mean corpuscular haemoglobin (MCH), and mean corpuscular haemoglobin concentration (MCHC) of the males were observed to be 851.31 ± 27.12 *μ*³, 100.05 ± 3.66 pg, and 11.74 ± 0.17%, respectively. In females the mean values of MCV, MCH, and MCHC were 822.41 ± 13.31 *μ*³, 96.71 ± 6.90 pg, and 11.75 ± 0.73%, respectively. However, the differences between the means of the corpuscular values were not significant (Table 1). In *Polypedates maculatus, *percentage of hemoglobin, MCV, and MCHC showed significant differences between male and female frogs [8].

### 3.4. WBC Count, Differential Leukocyte Count, and Neutrophil to Lymphocyte Ratio

More WBCs were observed in females than in males (Table 1). As mean WBC count was 12.12 × 10³±0.33/mm^3^ in males and 12.13 × 10³±0.69/mm^3^ in females, the difference was not significant (Table 1). However, percentage of lymphocytes, monocytes, eosinophils, and basophils remained higher in males than in females. But, the percentage of neutrophils was higher in females than in males (Table 1). The difference was significant only for neutrophils (*t* = 3.535, df = 6, *P* = 0.012).

The neutrophil to lymphocyte (N/L) ratio in females (0.48 ± 0.02) was more than in males (0.42 ± 0.03) (Table 1) and the difference was found to be significant (*t* = 3.328, df = 6, *P* = 0.058). According to Davis [36], the average reference range of amphibian N/L ratios falls within 0.01 to 0.67 and the N/L ratio of the present study falls within this range. There exists a close link between leucocyte profiles and glucocorticoid levels. Specifically, these hormones act to increase the percentage of neutrophils while decreasing the percentage of lymphocytes. This phenomenon is seen in all the five vertebrate taxa, namely, pisces, amphibia, reptilia, aves, and mammals, in response to either natural stressors or exogenous administration of stress hormones [37]. For ecologists, therefore, high ratio of neutrophil to lymphocytes in blood samples reliably indicates high glucocorticoid levels. Furthermore, this close relationship between stress hormones and N/L ratio needs to be highlighted more prominently in hematological assessments of stress for a better interpretation of results [37].

### 3.5. Serum Biochemical Parameters

The levels of blood serum protein, cholesterol, glucose, urea, and uric acid were found to be higher in females than in males. However, the serum creatinine value was more in males than in females (Table 2). The difference was found to be significant for the levels of glucose (*t* = 3.70, df = 6, *P* = 0.010), urea (*t* = 14.04, df = 6, *P* = 0.0001), and creatinine (*t* = 3.32, df = 6, *P* = 0.016). No significant differences were observed for the level of protein, cholesterol, and uric acid in the blood within the sexes.

## 4. Conclusion

As the study represents the first hematological serum biochemical investigation of the Rhacophorid frog, *Polypedates teraiensis*, the values can serve as general reference values for future investigations involving this species and other anuran species. This study would be helpful to understand the relevance of the data described earlier for the tadpoles of this species.

## Figures and Tables

**Table 1:** Hematological parameters observed in adult males and females of *Polypedates teraiensis. *

Hematological parameters	Sex	
	Male	Female
	*X* ± SD (range)	*X* ± SD (range)
RBC count*	0.59 ± 0.01 (0.58–0.61)	0.62 ± 0.01 (0.61–0.65)
Haemoglobin conc. (%)	5.95 ± 0.12 (5.8–6.1)	5.82 ± 0.29 (5.5–6.2)
Packed cell volume (%)	50.62 ± 1.10 (49.5–52.0)	51.55 ± 0.95 (50.5–52.5)
Mean corpuscular volume (µ³)	851.31 ± 27.12 (819.67–866.66)	822.41 ± 13.31 (803.07–833.33)
Mean corpuscular haemoglobin (Pg)	100.05 ± 3.66 (96.72–105.17)	96.71 ± 6.90 (90.76–105.76)
Mean corpuscular haemoglobin conc. (%)	11.74 ± 0.17 (11.71–11.95)	11.75 ± 0.73 (11.3–12.77)
RBC, length (µm)*	16.87 ± 1.3 (15.5–18.5)	19.77 ± 1.53 (18.0–21.5)
RBC, breadth (µm)*	8.95 ± 0.12 (8.8–9.1)	8.62 ± 0.34 (8.2–8.9)
Length/breadth of RBC	1.88 ± 0.16 (1.77–2.10)	2.29 ± 0.18 (2.02–2.41)
Surface area of erythrocyte (µm²)	117.77 ± 9.2 (107.60–126.98)	153.01 ± 35.11 (121.52–191.35)
Length of RBC's nucleus (µm)	6.15 ± 0.26 (5.9–6.5)	7.5 ± 1.73 (5.9–9.5)
Breadth of RBC's nucleus (µm)	5.0 ± 0.08 (4.9–5.1)	5.5 ± 0.90 (4.5–6.7)
Length/breadth of RBC's nucleus	1.22 ± 0.04 (1.18–1.27)	1.35 ± 0.15 (1.16–1.52)
Surface area of RBC's nucleus (µm²)	23.98 ± 1.27 (23.01–25.85)	33.0 ± 12.80 (20.7–49.64)
WBC count (number of WBC/mm³)	12.12 ± 0.33 (11.8–12.5)	12.15 ± 0.69 (11.5–12.9)
Neutrophil (%)*	23.52 ± 1.30 (22.0–25.0)	26.66 ± 1.16 (25.0–27.6)
Lymphocyte (%)	55.52 ± 2.04 (53.1–58.0)	54.92 ± 0.47 (54.6–55.6)
Basophil (%)	3.5 ± 0.24 (3.2–3.8)	2.8 ± 1.1 (1.9–4.2)
Eosinophil (%)	11.02 ± 1.12 (10.0–12.5)	9.97 ± 0.17 (9.8–10.2)
Monocyte (%)	6.42 ± 0.51 (5.9–7.1)	5.7 ± 0.46 (5.0–6.0)
Neutrophil/lymphocyte ratio*	0.42 ± 0.03 (0.37–0.45)	0.48 ± 0.02 (0.45–0.50)
Diameter of neutrophil (µm)	10.5 ± 0.55 (9.0–11.5)	10.5 ± 0.45 (10.0–11.5)
Area occupied by neutrophil (µm²)	87.54 ± 7.95 (63.62–103.81)	87.54 ± 9.75 (78.55–103.81)
Diameter of large lymphocyte (µm)*	9.5 ± 0.23 (7.5–11.5)	9.2 ± 0.35 (7.5–12.0)
Area occupied by large lymphocyte (µm²)	70.84 ± 6.32 (44.15–121.31)	66.44 ± 6.5 (44.15–113.04)
Diameter of small lymphocyte (µm)	5.25 ± 0.31 (4.0–6.0)	5.1 ± 0.65 (4.1–5.9)
Area occupied by small lymphocyte (µm²)	21.43 ± 5.65 (12.56–28.26)	20.01 ± 5.98 (13.19–27.32)
Diameter of basophil (µm)	11.0 ± 0.58 (9.0–13.5)	10.5 ± 0.65 (9.5–13.5)
Area occupied by basophil (µm²)*	94.98 ± 5.9 (63.58–143.06)	86.54 ± 9.75 (70.84–143.06)
Diameter of eosinophil (µm)*	11.5 ± 3.81 (10.0–12.0)	12.0 ± 0.31 (9.5–13.5)
Area occupied by eosinophil (µm²)	103.81 ± 12.3 (78.50–113.04)	113.04 ± 8.99 (70.84–143.06)
Diameter of monocyte (µm)	11.5 ± 0.85 (10.0–12.0)	11.2 ± 0.56 (10.0–12.0)
Area occupied by monocyte (µm²)	103.81 ± 10.32 (78.50–113.04)	98.47 ± 9.5 (78.50–113.04)

*X*: mean; SD: standard deviation; Pg: pictogram. *Difference in values between males and females statistically significant.

**Table 2:** Serum biochemical parameters observed in adult males and females of *Polypedates teraiensis. *

Serum biochemical parameters	Sex	
	Male	Female
	*X* ± SD (range)	*X* ± SD (range)
Protein (g/dL)	11.8 ± 0.54 (11.2–12.4)	12.75 ± 0.53 (12.3–13.5)
Cholesterol (g/dL)	2.0 ± 0.08 (1.9–2.1)	2.17 ± 0.09 (2.1–2.3)
Glucose (g/dL)^a^	50.75 ± 5.37 (44.0–56.0)	64.5 ± 5.25 (60.0–72.0)
Urea (g/dL)^a^	71.25 ± 0.95 (70.0–72.0)	80.0 ± 0.81 (79.0–81.0)
Uric acid (g/dL)	4.95 ± 0.31 (4.5–5.2)	5.15 ± 0.12 (5.0–5.3)
Creatinine (g/dL)^a^	3.1 ± 0.08 (3.0–3.2)	2.92 ± 0.09 (2.8–3.0)

*X*: mean; SD: standard deviation. ^a^Difference in values between males and females statistically significant.
